# Surgeon Preferences and Outcome Predictions for Displaced Distal Radius Fractures in Elderly Patients: A Survey of 151 Surgeons Making 4,832 Treatment Predictions Based on Patient Profiles and Radiographs

**DOI:** 10.1016/j.jhsg.2026.101030

**Published:** 2026-05-20

**Authors:** Nina Burgert, Dirk P. ter Meulen, Niels W.L. Schep, Joost W. Colaris, Henk A. Formijne Jonkers, Nienke W. Willigenburg, Rudolf W. Poolman, DART Surgeon Survey Study Group, Thomas P.A. Baltes, Thomas D. Berendes, Christiaan J.A. van Bergen, Arne Berger, Michiel E.R. Bongers, Liesbeth E.A. Boonstra, Tim A.E.J. Boymans, Bart ten Brinke, Pieter H.J. Bullens, Michaël P.A. Bus, M. Dietvorst, Pim A.D. van Dijk, A.J. Dijkstra, W.J. van Doorn, J. Duijff, Koen Dullaert, Jan Paul Frölke, Jasper G. Gerbers, J.C. Goslings, R.J. Hillen, J. Hoffman, Jochem M. Hoogendoorn, Stein J. Janssen, Louis de Jong, Pishtiwan Kalmet, Diederik H. Kempen, M.P. van de Kerkhove, Mark H.F. Keulen, Arthur J. Kievit, Nick J.H.M. In den Kleef, Sander Koëter, Joost TP. Kortlever, Tim Kraal, Pepijn Krielen, Niels Laas, Bart Lambert, Jelle P. van der List, Jan K.G. Louwerens, Claudia Lowik, Oscar E.C. van Maarseveen, Geert Meermans, Duncan E. Meuffels, Matthijs V. Nijenhuis, R. Onstenk, Ante Prkic, T.P. Saltzherr, Matthias Schafroth, Bernard Schutte, Charlie Sewalt, Lysanne van Silfhout, Malou E. Slichter, Diederik P.J. Smeeing, M.P. Somford, W.R. Spanjersberg, Willemijn Spierenburg, Charles Stevens, Nicky Stoop, Teun Teunis, Lucas Timmermans, Jaap J. Tolk, Clinton van Trikt, Kamil van der Velde, Ronald A.W. Verhagen, J. Vermeulen, Anne J.H. Vochteloo, David Visser, Luuk de Wert, Pieter B. de Witte, Lisa Worner, Rutger G. Zuurmond, P. van der Zwaal

**Affiliations:** ∗ Department of Orthopedic Surgery, OLVG Hospital, Joint Research, Amsterdam, the Netherlands; † Department of Trauma Surgery, Maasstad Hospital, Rotterdam, The Netherlands; ‡ Department of Orthopaedics and Sports Medicine, Erasmus University Medical Center, Rotterdam, the Netherlands; § Department of Trauma Surgery, OLVG Hospital, Amsterdam, the Netherlands; ¶ Department of Orthopedic Surgery, Leiden University Medical Center, Leiden, the Netherlands

**Keywords:** Distal radius fracture, Elderly, Outcome prediction, Surgeon decision-making, Treatment preferences

## Abstract

**Purpose:**

Vital elderly patients with substantially displaced intra-articular distal radius fractures often undergo surgical treatment, while those with notable comorbidities or less severe fractures are generally treated nonsurgically. As guidelines leave room for interpretation, surgeons must rely on intuition and experience when making treatment decisions. This study explores surgeons’ treatment preferences, expectations regarding outcomes, and their ability to predict functional outcomes.

**Methods:**

An online survey was conducted among Dutch trauma and orthopedic surgeons. Participants evaluated 16 patient profiles derived from a randomized controlled trial comparing surgery with cast immobilization. Each profile included patient characteristics and radiographs. Surgeons, blinded to the actual treatments and outcomes, indicated their treatment preference and predicted the expected outcomes for both treatment options.

**Results:**

A total of 151 surgeons made 4,832 treatment outcome predictions. Participants generally preferred surgical treatment for certain patient profiles and expected more favorable outcomes after surgery than after cast immobilization. However, only 51.5% of outcome predictions corresponding to the actual treatment were correct. No significant differences in predictive accuracy were observed between hand and wrist specialists, other consultants(not specifically hand and wrist specialists), and residents.

**Conclusions:**

Orthopedic and trauma surgeons, regardless of subspecialty or experience, demonstrated limited ability to predict functional outcomes for elderly patients with displaced distal radius fractures based on written information and radiographs. With only 51.5% of predictions correct, accuracy did not meaningfully exceed chance. Surgeons generally favored surgical treatment and expected better outcomes than with casting.

**Type of study/level of evidence:**

Prognostic V.

Distal radius fractures are the second most common type of fractures in the elderly, aged 65 year or older, following hip fractures.^1^ As the population ages, the incidence of distal radius fractures is expected to increase, posing a substantial burden on health care expenditures.^2^^,^^3^

Patients with distal radius fractures may undergo either conservative treatment or surgical treatment. Conservative treatment typically consists of closed reduction followed by casting and is considered safe and cost effective.^4^, ^5^, ^6^ However, reduction is not always successful, and even successfully reduced fractures can redisplace. Both scenarios result in malunion, which may lead to pain and loss of function. However, in elderly patients, even a malunion often leads to a satisfactory function.^7^, ^8^, ^9^ Furthermore, radiographic alignment does not seem to be related to function.^10^

Open reduction and internal fixation (ORIF) is the most commonly used surgical technique. Its benefits include anatomical restoration and stable fixation, which allow for early mobilization and may lead to a faster recovery. In elderly patients, early mobilization may help maintain independence and may reduce the need for additional care, such as home assistance or physical therapy. However, surgical treatment also carries disadvantages, including the risk of complications and higher costs.^4^, ^5^, ^6^

Several studies have identified factors influencing surgeons’ choice between surgical and conservative treatment for distal radius fractures.^11^^,^^12^ These decisions are typically based on a combination of fracture characteristics and patient-related factors such as age, comorbidities, and functional status. Greater fracture severity, including dorsal tilt or complex intra-articular patterns such as AO type C fractures, is often associated with surgical treatment. At the same time, multiple comorbidities tend to result in a preference for nonsurgical management.^11^^,^^12^

Although these studies provide insight into which factors surgeons consider when recommending treatment, they do not evaluate whether surgeons can predict the outcomes of their preferred treatments. The current study, therefore, aimed to assess whether orthopedic and trauma surgeons can predict functional outcomes after surgical and cast treatment in elderly patients with displaced distal radius fractures, based on patient profiles and radiographs.

## Methods

This online survey was conducted between May and September 2023 among orthopedic surgeons, trauma surgeons, and residents in the Netherlands. Surgeons from the Dutch Orthopedic Association and the Dutch Society for Trauma Surgery were invited to participate. To be eligible, surgeons had to treat at least 10 distal radius fractures a year. This survey was built in Castor EDC. The first invitation was sent on June 21, 2023. The study was registered at clinicaltrials.gov (NTC05942950).

## Ethical Considerations

This study was approved by the local Advisory Committee for Scientific Research, number WO 23.056. The front page of the survey contained an information letter and an informed consent form. Participants were informed about the study and storing of their data. Because participating in the survey required a substantial effort from the surgeons, we offered group authorship to all participants who completed the survey.

## Background of Participants

Participants were first asked to provide background information, including their age, years of experience as attending surgeons, and any subspecialty training.

## Patient Profiles

The survey continued with 16 patient profiles, selected from the DART study.^13^ The DART study is a multicenter randomized trial in the Netherlands. It evaluated whether cast treatment is noninferior to surgical treatment for elderly patients aged 65 years or older with substantially displaced intra-articular distal radius fractures. The DART study—and therefore also this survey—only included patients with intra-articular fractures and radiological fracture characteristics after closed reduction that were not acceptable according to the Dutch guideline.^14^ According to the Dutch guidelines for distal radius fractures, a fracture position is nonacceptable when one or more of the following criteria are met: radial inclination of ≤15°, radial height ≤5 mm, dorsal tilt >15°, volar tilt >20°, or an intra-articular gap or step-off ≥2 mm.

Wrist function was measured with the Patient-Rated Wrist Evaluation (PRWE).^15^ The PRWE is a patient-reported outcome measurement that evaluates wrist function and has 15 questions. The total score ranges from 0 to 100, 0 indicating a perfect function and 100 representing the worst function possible. The 16 patient profiles were selected from the DART study population based on treatment outcome in PRWE. We selected the most “extreme” outcomes, that is, the patients with the worst and best improvement in PRWE from both treatment groups. These “extreme” outcomes were chosen because these should be easier and relevant to discriminate than patients with outcomes close to the average.

Eight profiles represented patients who underwent surgery (ORIF), either with a volar and/or dorsal plate, of whom four had the best outcomes and four had the worst outcomes. The other eight profiles represented patients who underwent cast treatment; again, four patients with the best and four with the worst outcomes were selected. To determine the best and worst outcomes, we used the difference in PRWE (Δ PRWE) between the PRWE score pretrauma and at 1-year follow-up. PRWE score pretrauma was obtained through a questionnaire completed immediately after the trauma, ensuring that patients still recalled their pretrauma status. Patients with a good outcome had the smallest Δ PRWE, that is, recovery (close) to their pretrauma status. Patients with a poor outcome had the largest Δ PRWE.

### Characteristics of patient profiles

Each of the 16 patient profiles contained the following characteristics: sex, age, dominant hand, smoking, diabetes, corticosteroid use, PRWE score before trauma, radiographs, and the Groningen frailty indicator score.^16^ The Groningen frailty indicator is a validated questionnaire to assess the vulnerability of older adults by evaluating different aspects of health and functioning. The score ranges from 0 to 15. Patients who score 4 or higher are considered frail. It contains questions such as “Are you able to do the grocery shopping completely independently?.”

**Figure 1:**
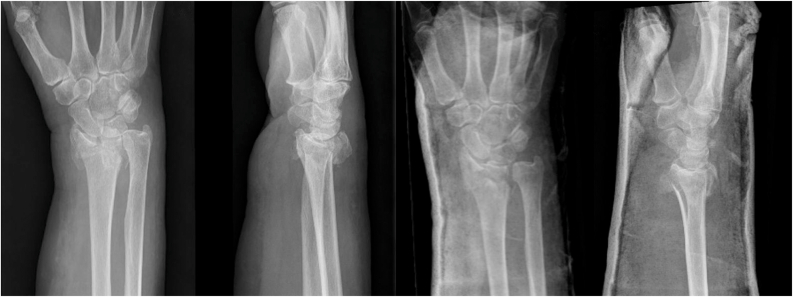
Example of patient profile.

Radiographs included posterior-anterior and lateral views before and after reduction. An example of a patient profile is presented in Figure 1. Surgeons were unaware of the fact that the patient profiles in the survey were extracted from the DART study. Surgeons were not informed about the treatment that patients had received, nor about the outcome of this treatment (Δ PRWE).

**Table:** 

Sex:	Female
Age:	71 years
Dominant hand:	No
Smoking:	No
Diabetes:	No
Corticosteroid use:	No
Groningen Frailty Indicator:	5 (frail patient)
PRWE before fracture:	0
1 Which treatment (surgery (ORIF) or casting) do you think will have the best outcome?	
Surgery (ORIF)	
Cast treatment	
We ask you to express the effect of both surgical treatment and cast treatment as a change in wrist function. The change is measured with the PRWE before and 1 year after trauma. (The PRWE scale ranges from 0 to 100, with 0 indicating a perfect function and 100 representing the worst possible function.)	
2 What effect do you expect from surgery (ORIF) when comparing the PRWE before trauma with the PRWE 1 year after trauma?	
Good outcome (0 (same as preinjury level) – 11.5 points difference in PRWE)	
Moderate outcome (11.5 – 30 points impairment in PRWE)	
Poor outcome (≥30 point impairment in PRWE)	
3 What effect do you expect from casting when comparing the PRWE before trauma with the PRWE 1 year after trauma?	
Good outcome (0 (same as preinjury level) – 11.5 points difference in PRWE)	
Moderate outcome (11.5 – 30 points impairment in PRWE)	
Poor outcome (≥30 point impairment in PRWE)	
There were 16 patient profiles, each accompanied by radiographs (before and after reduction) and relevant patient characteristics. For each profile, participants were asked to answer the three specified questions.

### Description of patient profile question

Three questions were asked for each patient profile: (1) whether the surgeon preferred ORIF or cast treatment for that patient; (2) what the expected outcome would be if the patient underwent ORIF; and (3) what the expected outcome would be if the patient underwent cast treatment.

The latter two outcomes were scored on a three-point Likert scale (good, moderate, poor). A good outcome was seen as no clinically relevant change, that is, a maximum 11.5 points worsening in Δ PRWE score compared to pretrauma. This value was chosen based on the minimal clinically important difference for the PRWE, which is 11.5.^17^ A moderate outcome was an impairment of 11.5 – 30 points on the Δ PRWE score compared to pretrauma, and a poor outcome was an impairment of ≥30 points on the Δ PRWE score compared to pretrauma. The cut-off point of 30 points for poor function was chosen arbitrarily, but set relatively high to facilitate distinction between patients who score well and patients who score poorly. An improvement in Δ PRWE was not expected, as the baseline PRWE score referred to the pretrauma state, and all patients had good wrist function at baseline.

## Outcome Measurements

### Preferred treatment

For each patient profile, participants were asked to indicate their preferred treatment, surgical (ORIF) or conservative (cast immobilization). This question aimed to capture respondents’ treatment preferences based on the provided clinical information and radiographs.

Treatment preferences were summarized descriptively as proportions. These preferences were not compared to the treatments that patients actually received, as the profiles were derived from a randomized controlled trial in which treatment allocation was determined by randomization rather than by clinical judgment. In other words, the surgeons’ responses in the survey were not compared with the treatment that was actually administered to the patient profiles.

### Expected outcome

Participants were asked to indicate their expected outcome for both surgical and cast treatment options. These responses reflect the surgeons’ perceived likelihood of success for each treatment.

Responses were recorded using a three-point Likert scale (good−moderate−poor outcome). For analysis, the expected outcomes were presented as the percentage of participants who anticipated a good outcome for each treatment. This outcome measure is independent of the actual clinical results and instead captures participants’ perceptions of effectiveness for each treatment based on the patient profiles.

### Correctly predicted treatment outcomes (primary outcome)

To calculate the percentage of patient profiles for which participants correctly predicted the actual treatment outcome, only half (n = 2,416) of the predicted outcomes could be used, that is, those corresponding to the treatment, the patient actually received during the randomized trial. Predictions regarding the alternative (nonreceived) treatment were excluded from the analysis, as the true outcome of that treatment is unknown.

Predicted outcomes were dichotomized: all predicted PRWE scores worse than 11.5 were categorized as poor outcomes, and scores of 11.5 or better were categorized as good outcomes. Correct predictions were defined as agreement between the predicted and actual outcome.

## Statistical Analysis

The preferred treatment and expected outcomes were analyzed descriptively. The percentage of correct predictions was first calculated per patient profile and then averaged across all profiles. 95% CI were calculated to reflect the reliability of the average estimate. Additionally, we compared prediction accuracy between patients with good and poor actual outcomes per treatment group with a Chi-square (χ^2^) test. To explore subgroup differences, respondents were divided by professional background. We compared the percentage of correct predictions between surgeons with and without a hand or wrist subspecialty (self-reported, as “hand surgeon” is not a protected title in the Netherlands), and between specialists and residents.

Statistical significance was defined as *P* < .05. All analyses were performed using SPSS version 29.0 (IBM Corp.).

## Results

### Participants

A total of 160 participants completed the full survey. However, nine participants were excluded because they did not treat at least 10 distal radius fractures a year, resulting in 151 participants. Characteristics of trauma- and orthopedic surgeons are presented in the Table 1.

**Table 1:** Characteristics of Survey Participants

Category	Subcategory	N (%)
Specialist	Total	96 (63.6)
	– Trauma surgeon	*37 (38.5)*
	– Hand surgeon	*59 (61.5)*
Resident	Total	55 (36.4)
	– Resident general surgery	*14 (25.5)*
	– Resident orthopedic surgery	*41 (74.5)*
Subspecialty∗	Trauma	52 (54.2)
	Hand/wrist	46 (47.9)
	Elbow	40 (41.7)
	Shoulder	40 (41.7)
	Back	6 (6.3)
	Hip	54 (56.3)
	Knee	43 (44.8)
	Ankle/foot	34 (35.4)
Years of experience specialist	0–5 years	32 (33.3)
	>5–10 years	24 (25.0)
	>10–15 years	25 (26.1)
	>15 years	15 (15.6)
Trauma time†	0–25%	31 (20.5)
	25–50%	66 (43.7)
	50–75%	21 (13.9)
	75–100%	33 (21.9)
Number of DRFs a year‡	10–25	27 (17.9)
	>25–50	50 (33.1)
	>50–99	22 (14.6)
	≥100	52 (34.4)
	Total	151 (100)

∗ Trauma is listed as a subspecialty only for orthopedic surgeons. All other subspecialties represent totals for both trauma and orthopedic surgeons. Participants could select multiple subspecialties.

† Trauma time refers to the percentage of total working time spent on trauma care.

‡ DRF refers to the number of patients treated per year, either conservatively or surgically.

#### Preferred treatment

For each separate patient profile the treatment preferences are presented in the Table 2. A few notable observations stand out from this table. Patient profile number 9 had a poor outcome after ORIF. ORIF was preferred by 6.6% of the surgeons, while 93.4% preferred casting for this case.

**Table 2:** Treatment Preferences and Predicted Results for All 16 Patient Profiles∗

Profile Group	Patient Profile	Δ PRWE	Surgeon Preferred ORIF % (95% CI)	Correctly Identified Outcome % (95% CI)
Good outcome to ORIF	4	−8.5	68.9	49.7
	5	0	43.7	27.2
	8	0	89.4	61.6
	10	−0.5	82.8	43.7
*Group average*		−*2.3 (*−*8.9* to *4.4)*	*71.2 (39.0−103.4)*	*45.6 (22.8−68.3)*
Poor outcome to ORIF	1	34	67.5	29.8
	9	60.5	6.6	42.4
	11	61	60.9	72.2
	15	60.5	33.1	66.2
*Group average*		*54.0 (32.8−75.2)*	*42.0 (−2.4−86.5)*	*52.7 (20.9−84.4)*
Good outcome to casting	3	−17	69.5	21.2
	6	−5.5	53	11.3
	13	0	42.4	18.5
	16	−1.5	31.1	36.4
*Group average*		*−6.0 (−18.2* to *6.2)*	*49.0 (23.0−75.0)*	*21.9 (5.0−38.7)*
Poor outcome to casting	2	51	86.1	94.7
	7	47.5	85.4	92.1
	12	51	21.9	64.9
	14	66.5	56.3	91.4
*Group average*		*54.0 (40.5−67.5)*	*62.4 (14.1−110.8)*	*85.3 (63.5−108.0)*
Total average			*56.2 (43.0−69.4)*	*51.5 (37.0−65.9)*

∗ Group averages are presented as means with 95% confidence intervals. Δ PRWE describes the change between the PRWE score pretrauma and 1 year after trauma. Note that patient profiles groups with a poor outcome have high PRWE values, as a high PRWE is indicative of a poor function.

**Figure 2:**
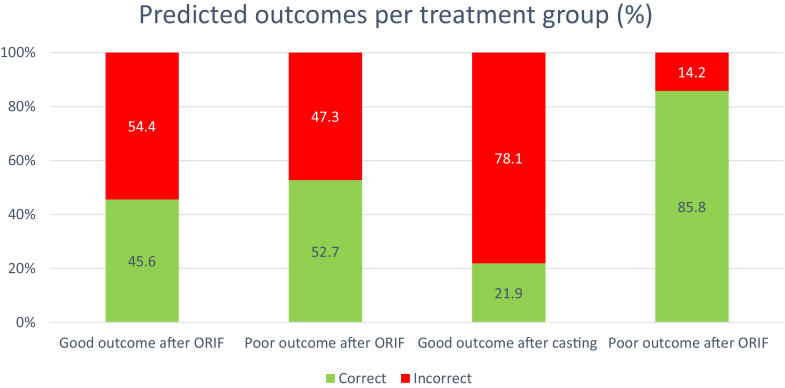
The treatment preferences per patient profile group. For example, the first bar represents the group of patients who responded positively to ORIF. Of the survey participants, 71.2% preferred ORIF for these patients.

Patient profile number 12 had a poor outcome following casting. For this case, 21.9% of the respondents preferred ORIF, and 78.1% preferred casting. The Figure 2 provides a visual representation of treatment preferences per patient profile group and not the correct/incorrect predictions. Surgeons preferred ORIF for 71.2% of the patient profiles in the “good outcome after ORIF” profile group, compared with 42.0% in the “poor outcome after ORIF” group, as shown in Figure 2. Furthermore, surgeons preferred ORIF for 49.0% of the patient profiles in the “good outcome after casting” group, compared with 62.4% of the patient profiles in the “poor outcome to casting” group.

#### Expectations

The Table 3 presents the distribution of expected outcomes for ORIF and casting. The total number of predictions was 4,832 (151 respondents × 16 patient profiles × 2 treatments). A good outcome after ORIF was expected in 46.0% of patients. A good outcome of casting was expected in 18.3% of patients. A poor outcome to ORIF was expected in 2.4% of surgically treated patients, compared with an expected poor outcome in 29.8% of patients treated with a cast.

**Table 3:** Expected Outcomes for Both Treatments∗

Treatment	Outcome	Frequency	%
ORIF	Good	1112	46
	Moderate	1247	51.6
	Poor	57	2.4
	Total	2416	100
Casting	Good	443	18.3
	Moderate	1252	51.8
	Poor	721	29.8
	Total	2416	99.9†

∗ The distribution of expected outcomes for ORIF and casting. A total of 4,832 responses were recorded (151 participants × 16 patient profiles × 2 treatments). Participants expressed more optimism about the expected outcome of ORIF compared to casting.

† Because of rounding, the percentages for casting do not add up to exactly 100%.

#### Correctly predicted treatment outcomes

**Figure 3:**
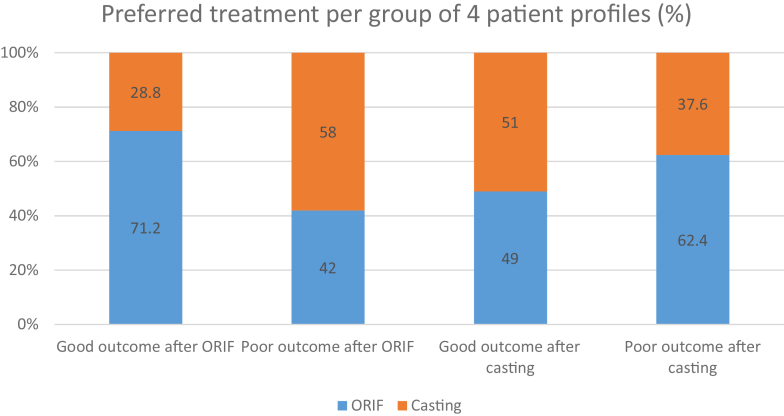
The percentage of correctly predicted outcomes per patient profile group. For example, the first bar represents the group of patients who responded positively to ORIF. Of the survey participants, 45.6% accurately predicted this correctly, while 54.4% incorrectly predicted that this group would not recover well from ORIF.

The Table 2 presents the percentage of correctly predicted outcomes for each of the 16 patient profiles. A total of 2,416 predictions (151 respondents × 16 patient profiles) were made, and the overall percentage of correct predictions was 51.5% (95% CI, 37.0−65.9). For example, 49.7% of the respondents correctly predicted that patient profile 4 would have a good outcome after surgery. For clarity, the predictions about the effect of treatment that the patient did not receive were disregarded because that outcome is unknown. Figure 3 visually presents the percentage of correct predictions per patient profile group, as assessed by survey participants.

The mean percentage of correct predictions of hand specialists was 52.0% (95% CI, 49.1−55.0) compared with 51.2% (95% CI, 49.4−53.0) for nonhand specialists (*P* = .386). The mean percentage of correct predictions of (orthopedic) trauma surgeons was 51.2% (95% CI, 49.3−53.1) compared with 51.5% (95% CI, 48.9−54.1) for residents (*P* = .561).

## Discussion

The results of this study indicate that, based on radiographs and written patient information, the surgeons were unable to accurately predict treatment outcomes for elderly patients with a distal radius fracture. The overall percentage of correct predictions was 51.5%, which is equivalent to a coin toss. Subgroup analyses comparing experienced hand surgeons with nonhand specialists and specialists with residents revealed similar accuracy rates, with no statistically significant differences between groups.

However, as illustrated in Figure 2, treatment preferences appeared to align somewhat with actual outcomes. Surgeons preferred ORIF in 71.2% of patients who had a good recovery following surgical treatment, compared to only 42.0% in those with a poor surgical outcome. In contrast, ORIF was preferred in 49.0% of patients who had a good outcome after casting, and in 62.4% of those with a poor casting outcome. While Figure 3 seems to suggest that surgeons can correctly identify the vast majority of patients with poor outcomes after casting, this coincided with only 21.9% correctly identified patients with good outcome after cast treatment.

Overall, surgeons expressed more optimistic expectations for surgical outcomes than for casting: averaged over all patient profiles, 46% good results were anticipated with ORIF, whereas only 18.3% good outcomes were anticipated with casting. The percentage of expected poor outcomes were 2.4% for ORIF and 29.8% for cast treatment. Despite this, ORIF was preferred in only 56.2% of all patient profiles.

## Comparison With Literature

Previous research has examined the factors that influence surgeons’ treatment decisions. Surgeons tend to prefer conservative treatment for patients with higher comorbidity scores, while more severely displaced fractures or AO type C fractures are more often treated surgically.^12^ Similarly, another study found that age and fracture characteristics, particularly angulation, were the most influential factors in surgical decision-making.^11^ Notably, these variables were presented in our patient profiles. Paradoxically, radiographs may offer limited value in guiding treatment decisions. Several studies, including a recent one by Lawson et al,^10^ have shown a poor correlation between radiographic appearance and functional outcomes. Therefore, to better predict which patients should receive surgical treatment, more emphasis may need to be placed on factors such as frailty, comorbidities, or activity level, as highlighted in the study by Jayaram et al.^18^ Alternatively, incorporating additional elements such as patient preferences in a decision aid might improve individualized decision-making.^19^

## Strengths and Limitations

A major strength of this study is the use of real patient data derived from a randomized clinical trial. This allowed us to avoid constructing hypothetical cases and instead base our findings on actual clinical scenarios. Furthermore, we deliberately selected the best- and worst-performing patients from each treatment group (ORIF and casting) to create maximum contrast between profiles with good and poor outcomes. We believe that this approach, maximizing the difference in outcome extremes, should facilitate the decision-making process. If surgeons are unable to accurately predict outcomes in such clearly contrasting cases, it is even less likely that they would succeed in predicting more subtle variations in clinical outcomes.

Several limitations of the study should also be acknowledged. First, the survey included an equal number of profiles with good and poor outcomes (four for each treatment), which does not reflect typical clinical practice where the majority of patients tend to have favorable outcomes. This artificial balance may have affected participants’ expectations and decision-making.

Second, the outcome predictions were based on PRWE scores, which are difficult to estimate based solely on written patient information and radiographs. As discussed earlier, radiographic findings are known to correlate poorly with final functional outcomes. Moreover, translating a real clinical presentation into a numerical PRWE estimate over a 1-year period is probably very challenging for any clinician.

Third, the study is subject to potential selection bias. The relatively high proportion of hand surgeons among respondents (47.9%) may not accurately reflect the broader population of surgeons treating distal radius fractures and could influence generalizability. It is however unlikely that the broader population of surgeons would be better in estimating treatment outcomes.

Fourth, complications can have a substantial impact on treatment outcomes and are often difficult to predict. In our study, complications were present in three of the selected patient profiles. For two of the surgically treated patients with poor outcomes a complication was registered. One required surgical denervation of the posterior interosseous nerve because of persistent pain, which may not be considered a complication since it is a results of symptoms rather than a cause. Another underwent hardware removal because of severe dorsal redisplacement in spite of surgical fixation which resulted in the flexor tendons contacting the volar plate. In the casting group, one patient developed a transient superficial radial nerve neuropathy but ultimately made a full recovery and was actually part of the patient profile group with a good outcome after cast treatment.

Fifth, it may be more difficult to predict treatment outcomes based solely on written patient profiles and radiographs, without actual contact with the patient. Ideally, clinical decision-making includes face-to-face interaction between patient and clinician to discuss treatment options and incorporate the patient’s preferences. Notably, several participating surgeons reported that they missed knowing the patient’s treatment preference, activity level and hobbies, which they consider important factors in the decision-making process. This information was unavailable in our study because the patient profiles were derived from a randomized controlled trial.

Our study demonstrates that orthopedic and trauma surgeons, regardless of subspecialty or level of experience, have a limited ability to predict functional outcomes for elderly patients with displaced distal radius fractures based on written patient information and radiographs. Although treatment preferences often favored surgical treatment, overall, only 51.5% of outcome predictions were correct. No significant differences in prediction accuracy were observed between hand and wrist specialists, other consultants (not specifically hand and wrist specialists), and residents. Surgeons generally expected more favorable outcomes following surgical treatment compared to cast immobilization.

## Conflicts of Interest

No benefits in any form have been received or will be received related directly to this article.
